# Drug–drug synthetic lethality to combat cancer: From bench to bedside

**DOI:** 10.1002/ctm2.1269

**Published:** 2023-05-23

**Authors:** Yu Chen, Wu Yang, Jiaojiao Zheng, René Bernards, Haojie Jin

**Affiliations:** ^1^ State Key Laboratory of Oncogenes and Related Genes Shanghai Cancer Institute Renji Hospital Shanghai Jiao Tong University School of Medicine Shanghai China; ^2^ Division of Molecular Carcinogenesis Oncode Institute The Netherlands Cancer Institute Amsterdam The Netherlands

**Keywords:** Cancer therapy

## 1 SYNTHETIC LETHALITY

Synthetic lethality, a term coined by Theodosius Dobzhansky in 1946,^1^ arises when co‐occurring mutations in two genes kill cells, whereas mutation of either alone is compatible with viability. Decades later, the concept of synthetic lethality was applied to cancer research, eventually leading to the approval of new therapies.^2^ One of the best‐known examples of this concept is the selective sensitivity of Breast Cancer Susceptibility Gene (BRCA)‐mutated cancers to poly (ADP‐ribose) polymerase (PARP) inhibitors. These BRCA mutant tumors are highly dependent on PARP‐mediated base excision repair of damaged DNA—a weakness exploited by PARP inhibitors. To date, PARP inhibitors have been approved for the clinical treatment of ovarian, breast, prostate and pancreatic cancers associated with BRCA1/2 mutations.^3^, ^4^

## 2 DRUG–DRUG SYNTHETIC LETHALITY

More recently, drug–drug synthetic lethality was developed to counter intrinsic drug resistance that often results from feedback‐mediated activation of the same or a parallel signalling pathway upon drug treatment. The high‐throughput technologies of RNAi (RNA interference, enabling downregulation of the expression of specific genes) and CRISPR‐Cas9‐mediated gene knockout have greatly facilitated unbiased discovery of such drug–drug synthetic lethality. Here, we will discuss two examples of drug–drug synthetic lethality, which have been translated from ‘bench to bed’, highlighting the power of high‐throughput screening in guiding the design of clinical combination therapies.

## 3 SHRNA‐BASED GENETIC SCREEN: COMBINATION OF BRAF INHIBITOR AND EGFR INHIBITOR IN COLORECTAL CANCER

In the early twenty‐first century, RNAi technology was introduced, allowing high‐throughput screening in cancer cells driven by specific oncogenic mutations. A particularly interesting example of the use of this technology relates to the proper therapy selection of different BRAF mutant cancers. Even though activating mutations in the BRAF oncogene (BRAF(V600E)) were found in some 70% of primary melanomas, some 10% of colorectal cancers and some 30%−70% of papillary thyroid carcinoma, clinical responses to the highly selective small‐molecule inhibitor of the BRAF(V600E) oncoprotein, vemurafenib (PLX4032), differed widely, ranging from a response rate of approximately 80% in melanoma to only 5% in BRAF mutant colorectal cancers (CRCs).

To investigate the molecular mechanism responsible for the intrinsic resistance of BRAF(V600E) CRCs to vemurafenib, Prahallad et al. performed a short hairpin RNA (shRNA)‐based genetic screen investigate whether inhibition of any of the 518 kinases in the human genome synergizes with BRAF(V600E) inhibition.^5^ It was found that BRAF inhibition caused rapid feedback activation of MAP kinase signaling through activation of Epidermal growth factor receptor (EGFR). BRAF inhibition in CRC cells exposed a sensitivity to the concomitant loss or inhibition of the receptor tyrosine kinase EGFR, both in vitro and in vivo. However, melanoma cells express little EGFR; therefore, BRAF inhibitors did not stimulate EGFR activation. These findings resulted in the approval by Food and Drug Administration (FDA) and European Medicines Agency (EMA) of combination treatment with the BRAF inhibitor encorafenib and the EGFR‐targeting antibody cetuximab for BRAF‐mutant metastatic colorectal cancers in 2020.^6^, ^7^ Not surprisingly, translation of these findings into clinical trials, and finally to FDA‐approval has taken 8 years. Given that the clinical trials were all performed in record time, this is a stark reminder that the trip from bench to bedside is long, even in the best possible scenario.

## 4 CRISPR‐BASED GENETIC SCREEN: COMBINATION OF EGFR INHIBITOR AND LENVATINIB IN LIVER CANCER

RNAi technology is prone to off‐target effects, meaning that RNAi targeting of one gene may accidentally co‐target another gene. Luckily, not long after RNA interference, the CRISPR gene editing technology was discovered, which can perfectly overcome the off target issues of RNAi technology and improve efficiency and accuracy.

In 2021, our group discovered a novel rational combination for the treatment of liver cancer in preclinical investigations. The pre‐clinical data were supported by encouraging evidence of clinical benefit in a proof‐of‐concept trial. The multi‐kinase inhibitor lenvatinib has been approved by the FDA for the first‐line treatment of unresectable hepatocellular carcinoma (HCC), but the overall response rate is only about 24%.^8^ Thus, in our study, we adopted a CRISPR‐based genetic screening test to dissect the mechanism of intrinsic resistance to lenvatinib seen in some three quarters of HCC patients. Through a CRISPR‐based synthetic lethality screen, we discovered a synergistic effect of combining an EGFR inhibitor with lenvatinib in multiple HCC cell lines that expressed high levels of EGFR. Biochemical studies revealed that inhibiting FGFR with lenvatinib led to the activation of the EGFR‐PAK2‐ERK5 pathway. Consequently, EGFR inhibition prevented the necessary feedback to activate these kinases, which inhibited the phosphorylation of MAP kinases. Furthermore, the combination of lenvatinib and EGFR inhibitors showed an efficient anti‐tumor effect in multiple in vivo HCC models that expressed high levels of EGFR. These HCC models included a cell‐line derived HCC xenograft model, a genetically engineered mouse model with orthotopic liver cancer, and an HCC PDX model. Based on these preclinical findings, we registered a phase I clinical trial (trial identifier NCT04642547) to investigate the safety and therapeutic efficacy of lenvatinib combined with gefitinib in patients with lenvatinib‐resistant HCC. The use of biomarkers can assist in the selection of appropriate patients for trials of combination therapies. Therefore, according to our preclinical observations, we only recruited patients with HCC that showed high EGFR expression levels for the study. Thus, among 12 patients with advanced HCC that were unresponsive to lenvatinib mono‐therapy, we found that four patients showed a confirmed partial response to the combination lenvatinib plus gefitinib therapy.

## 5 FUTURE OUTLOOK

**FIGURE 1:**
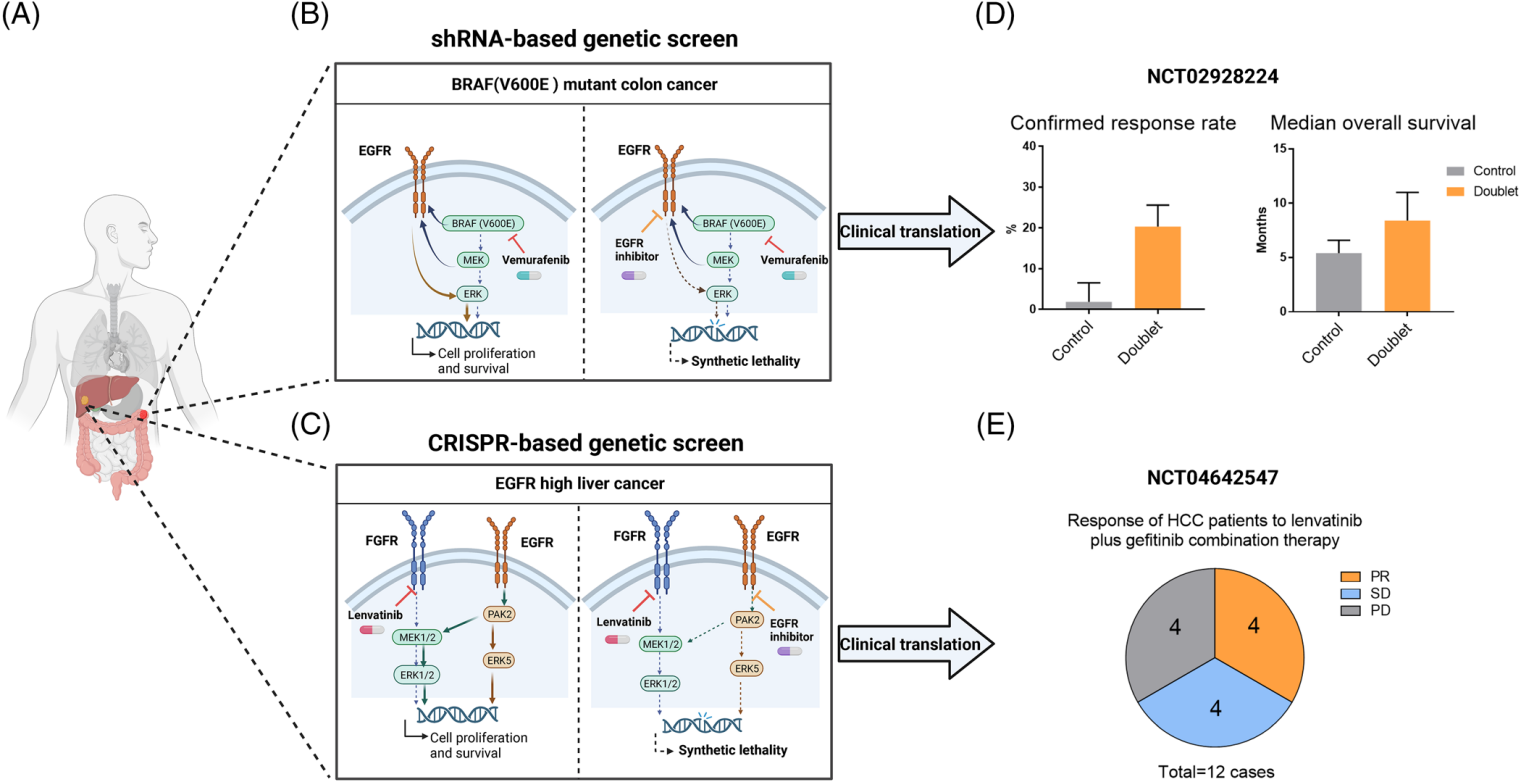
Drug–drug synthetic lethality against cancer: from bench to bedside. (A) Liver cancer and colorectal cancer are leading cause of cancer mortality worldwide. (B) Model showing the synthetic lethal interplay between vemurafenib and EGFR inhibitor in CRC with BRAF(V600E). (C) Model showing the synthetic lethal interplay between lenvatinib and EGFR inhibitor in liver cancer expressing high EGFR. (D) Bar plot showing the confirmed response rate and the median overall survival in the doublet‐therapy group (treated with encorafenib plus cetuximab, N = 113) as compared with the control group (treated with Irinotecan or FOLFIR plus cetuximab, N = 107). Data are seen mean ± 95% CI. (E) Pie plot showing the clinical responses among patients with lenvatinib‐resistant HCC after combination with lenvatinib and gefitinib (N = 12). PR, partial response; SD, stable disease; PD, progressive disease.

Both studies emphasize the advantage of using functional genetic screens to discover novel and effective drug–drug synthetic lethality interactions to improve the efficacy of targeted therapies (See Figure 1). Although genetic screening is very efficient for potential targets of ‘synthetic lethality’, many genes are undruggable, making compound screens more practical in some cases. Using a library of FDA‐approved compounds in combination with drugs that already in clinical use, yet have poor efficacy, may lead to some unexpected potential treatment suggestions.^9^

Furthermore, the use of two drugs may still not be enough. Despite combinatorial targeted therapies based on the latest understanding of signaling circuitry,^5^ patients with BRAF(V600E) mutant CRC only show modest clinical benefit from the BRAF plus EGFR inhibitor combination. Using a high‐throughput kinase activity mapping platform, Ruiz‐Saenz A et al identified that Proto‐oncogene tyrosine‐protein kinase (SRC) kinases are systematically activated in BRAF(V600E) CRC following targeted inhibition of BRAF and EGFR. Such studies can identify additional targets that need to be inhibited to obtain more lasting clinical responses in BRAF(V600E) CRC.^10^

Using combination therapies to treat cancer may seem simple in theory, but difficult in practice because of the many possible combinations of the present arsenal of cancer drugs. Rational drug combinations that show strong synergy based on insights in how signaling pathways communicate are a logical path forward.^2^ Near‐term benefits to patients may come from focusing on finding better combinations of existing drugs rather than the current emphasis on finding more drug targets.

## FUNDING INFORMATION

The National Natural Science Foundation of China Grant Numbers: NSFC82222047 and NSFC82073039; The Program of Shanghai Academic/Technology Research Leader, Grant Number: 22XD1423100.

## CONFLICT OF INTEREST STATEMENT

The authors declare no conflict of interest.
